# *WHO Model list of essential medicines*: visions for the future

**DOI:** 10.2471/BLT.24.292359

**Published:** 2024-08-29

**Authors:** Thomas Piggott, Lorenzo Moja, Benedikt Huttner, Patrick Okwen, Mario Carlo B Raviglione, Tamara Kredo, Holger J Schünemann

**Affiliations:** a Department of Health Research Methods, Evidence, and Impact, McMaster University, Hamilton, Canada.; b Department of Health Products Policy and Standards, World Health Organization, Geneva, Switzerland.; c Department of Surveillance, Prevention and Control, World Health Organization, Geneva, Switzerland.; d Department of Public Health, The University of Bamenda, Bamenda, Cameroon.; e Centre for Multidisciplinary Research in Health Science, University of Milan, Milan, Italy.; f Health Systems Research Unit, South African Medical Research Council, Cape Town, South Africa.; g Clinical Epidemiology and Research Center, Humanitas University & Humanitas Research Hospital, Via Rita Levi Montalcini 4, 20090 Pieve Emanuele, Milan, Italy.

## Abstract

The first version of the *World Health Organization Model list of essential medicines* contained 186 medicines in 1977 and has evolved to include 502 medicines in 2023. Over time, different articles criticized the methods and process for decisions; however, the list holds global relevance as a model list to over 150 national lists. Given the global use of the model list, reflecting on its future role is imperative to understand how the list should evolve and respond to the needs of Member States. In 2023, the model list Expert Committee recommended the World Health Organization (WHO) to initiate a process to revise the procedures for updating the model list and the criteria guiding decisions. Here, we offer an agenda outlining priority areas and a vision for an authoritative model list. The main areas include improving transparency and trustworthiness of the recommendations; strengthening connection to national lists; and continuing the debate on the principles that should guide the model list, in particular the role of cost and price of essential medicines. These reflections are intended to support efforts ensuring the continued impact of this policy tool.

## Résumé

La première version de la *Liste modèle des médicaments essentiels de l'OMS*, publiée par l'Organisation mondiale de la Santé, répertoriait 186 médicaments en 1977 et s'est enrichie jusqu'à en inclure 502 en 2023. Au fil du temps, différents articles ont critiqué ses méthodes et processus décisionnels; toutefois, elle reste importante au niveau mondial, servant de référence à plus de 150 listes nationales. Compte tenu de l'usage international qui en est fait, il est impératif de réfléchir à sa fonction future afin de comprendre comment elle peut évoluer et répondre aux besoins des États Membres. En 2023, le Comité d'experts chargé de la liste modèle a recommandé à l'OMS de se lancer dans la révision des procédures de mise à jour de cette liste, ainsi que des critères appliqués aux décisions. Dans le présent document, nous proposons un programme qui définit les priorités et les perspectives d'avenir pour que la liste fasse autorité. Parmi les principaux domaines abordés figurent l'amélioration de la transparence et de la fiabilité des recommandations, le renforcement des liens avec les listes nationales et la poursuite du débat sur les principes qui devraient orienter la liste modèle, en particulier le rôle joué par les coûts et les prix des médicaments essentiels. Ces réflexions ont pour but de soutenir les efforts permettant à cet instrument politique de conserver son impact.

## Resumen

La primera versión de la *Lista Modelo OMS de Medicamentos Esenciales* contenía 186 medicamentos en 1977 y ha evolucionado hasta incluir 502 medicamentos en 2023. Con el tiempo, diferentes artículos criticaron los métodos y el proceso de decisión; sin embargo, la lista conserva su relevancia mundial como lista modelo para más de 150 listas nacionales. Dado el uso global de la lista modelo, es imperativo reflexionar sobre su función futura para comprender cómo debe evolucionar la lista y responder a las necesidades de los Estados Miembros. En 2023, el Comité de Expertos de la lista modelo recomendó a la OMS que iniciara un proceso para revisar los procedimientos de actualización de la lista modelo y los criterios que guían las decisiones. En este documento, se ofrece un programa en el que se describen las áreas prioritarias y la visión de una lista modelo oficial. Entre las principales áreas se incluyen la mejora de la transparencia y la fiabilidad de las recomendaciones, el refuerzo de la conexión con las listas nacionales y la continuación del debate sobre los principios que deben guiar la lista modelo, en particular sobre la función del coste y el precio de los medicamentos esenciales. El objetivo de estas reflexiones es apoyar los esfuerzos que aseguren el impacto continuado de esta herramienta política.

## ملخص

احتوت النسخة الأولى من القائمة النموذجية للأدوية الاساسية التابعة لمنظمة الصحة العالمية (WHO) على 186 دواءً في عام 1977، وتطورت لتشمل 502 دواءً في عام 2023. وبمرور الوقت، انتقدت مقالات مختلفة الطرق والعمليات المتبعة لاتخاذ القرارات؛ ومع ذلك، تحتفظ القائمة بمكانة عالمية باعتبارها قائمة نموذجية بالنسبة لأكثر من 150 قائمة وطنية. ونظرًا للرواج العالمي للقائمة النموذجية، فإن التفكير في دورها المستقبلي يُعد ضروريًا لفهم كيفية تطور القائمة، والاستجابة لاحتياجات الدول الأعضاء. في عام 2023، قامت لجنة الخبراء المعنية بالقائمة النموذجية، بتوصية منظمة الصحة العالمية ببدء عملية لمراجعة إجراءات تحديث القائمة النموذجية والمعايير التي ترشد القرارات. ونحن هنا نقدم جدول أعمال يوضح المجالات ذات الأولوية، فضلاً عن رؤية لقائمة نموذجية تكون موضع ثقة. وتشمل المجالات الرئيسية تحسين الشفافية وجدارة التوصيات بالثقة، وتعزيز الارتباط بالقوائم الوطنية ومواصلة المناقشة حول المبادئ التي يجب أن توجه القائمة النموذجية، وبخاصة دور تكلفة الأدوية الأساسية وسعرها. والهدف من هذه الأفكار هو دعم الجهود الرامية إلى ضمان التأثير المتواصل لهذه الأداة السياسية.

## 摘要

世界卫生组织 (WHO) 于 1977 年公布的第一版《WHO 基本药物标准清单》包含 186 种药物，发展至 2023 年，该清单已包含 502 种药物。随着时间的推移，出现了对相关决策方法和过程颇有微词的许多文章；尽管如此，作为 150 多个国家清单的标准清单，该清单始终具有全球相关性。鉴于标准清单的全球适用性，反思该清单的未来作用对于了解清单应如何发展并满足会员国的需求至关重要。2023 年，标准清单专家委员会建议 WHO 启动流程，以修订标准清单的更新程序和决策指导标准。我们在此提供了概述优先领域的议程，并表明了权威性标准清单相关愿景。主要领域包括提高建议的透明度和可信度，加强与国家清单之间的联系，继续基于指导标准清单的原则开展辩论，特别是基本药物的成本和价格的作用。这些反思旨在支持为确保该政策工具持续发挥作用所做的工作。

## Резюме

Первая версия *Примерного перечня основных лекарственных средств ВОЗ* в 1977 году содержала 186 препаратов, в 2023 году он включает 502 препарата. Со временем в различных статьях были подвергнуты критике методы и процесс принятия решений, однако список имеет глобальное значение, являясь образцом для более чем 150 национальных списков. Учитывая глобальное использование примерного перечня, необходимо проанализировать его будущую роль для понимания того, как перечня должен совершенствоваться и отвечать потребностям государств-членов. В 2023 году Экспертный комитет по примерному перечню рекомендовал ВОЗ начать процесс пересмотра процедур обновления данного перечня и критериев, которыми руководствуются при принятии решений. В этой статье представлена программа действий, определяющая приоритетные области и видение авторитетного примерного перечня. К основным направлениям относятся: повышение прозрачности и достоверности рекомендаций, укрепление связи с национальными списками и продолжение дискуссии о принципах, которыми следует руководствоваться при составлении примерного перечня, в частности о роли стоимости и цены основных лекарственных средств. Приведенные размышления направлены на поддержку усилий, обеспечивающих постоянное воздействие этого средства осуществления политики.

## Introduction

The *World Health Organization model list of essential medicines* is almost 50 years old. The model list is a normative product of the World Health Organization (WHO), aimed at defining the medicines that represent the best value for patient needs globally to prioritize and support access, and therefore ultimately, health equity.^1^ Moreover, the model list is the policy tool that WHO shares with Member States as a model for best practices and developing processes for selecting essential medicines at the national level. While the model list is maintained by WHO and informed by appointed experts, its adoption as a standard for universal health coverage (UHC) is a national policy decision. The model list has evolved from including 186 medicines in 1977 to 502 in 2023, keeping pace with the expansion in diseases that have available treatments and the number of therapeutic products marketed.^2^^,^^3^ The current process to update the model list was agreed by Member States in 2001.^1^ However, with more medicines available and listed, decision-making for defining what is still essential today is increasingly complex.

The concept of what is an essential medicine has also evolved over time.^4^ The model list started as a parsimonious list of medicines that are considered of utmost importance, basic, indispensable and necessary for the health needs of the population, and included mostly small molecules.^2^ The approach to focus on only the safest, effective and high-value medicines is still a core feature of the model list, but today candidates for selection include biotechnology and complex molecules, radiopharmaceuticals and cell or gene therapies, among others. Removal of medicines from the model list when they are superseded by safer, more effective and better value alternatives is also needed. The model list is now also integrated with other health decision-making paradigms, including connections with health guidelines as well as access initiatives such as the Global Fund to Fight AIDS, Tuberculosis and Malaria, WHO Prequalification of Medical Products and the Medicine Patent Pool.^5^^–^^7^

Currently, the WHO Expert Committee on Selection and Use of Essential Medicines, a group of independent, international experts appointed by the WHO Director-General, updates the model list biennially. The experts recommend adding or removing medicines based on applications received in two-year cycles. Once adopted by the WHO Director-General, these recommendations become formal WHO recommendations to Member States. Applications can come from WHO departments or external applicants such as academia or pharmaceutical companies. The number of applications per cycle has increased, peaking at nearly 90 applications in 2021.^8^

Drawing on our previous work on the existing approach to the model list decision-making process,^9^^–^^12^ we here reflect on strengths and challenges over the past 50 years, and present a vision for where we believe the model list should head into the future.

## Successes of the model list

The WHO model list has served as a reference model list for many countries, with over 150 countries having created their own, national ones. Work examining the alignment of the WHO model list and national lists has been extensive, and areas of divergence have emerged.^12^^,^^13^ Nonetheless, several studies consider that the continued relevance, updating and support for making and implementing national essential medicine decisions are a success story in prioritizing medicines globally.^14^^,^^15^ A prominent example is human immunodeficiency virus (HIV). In the early 2000s, the model list listing of antiretroviral drugs, along with simultaneous prescribing guidelines by WHO, provided an authoritative voice recommending antiretroviral drugs, despite their high price.^16^ The listing of antiretrovirals was just one of the critical steps that contributed to improving access to these medicines. Not recommending antiretrovirals in the model list could have been an argument for not engaging in policies to reduce antiretroviral prices and would have further delayed access to treatment. The simultaneous communities’ advocacy for support to access policies was a major factor in raising policy-makers’ awareness of the addition of antiretrovirals in the model list and then national lists. 

More recently, the model list’s relationship to other decision-making tools has emerged, for example in relation to cancer medicines. In 2015, the first biologic therapies against solid and blood cancers, trastuzumab and rituximab antibodies, were included on the model list.^17^ These antibodies were later the first biologic therapies against cancers recommended on national lists, and in 2019 they were the first cancer biologic therapies that WHO prequalified.^18^ Evidence now exists showing that the availability of these therapies has increased in low- and middle-income countries.^19^^,^^20^ Notably, corresponding WHO guidelines and active voluntary licensing schemes for these medicines are absent.

However, evidence correlating the WHO model list and national derivatives to increased access is limited, indirect and speculative; no quantifiable effect on increased access to medicines is available. This challenge partly arises from assessing the model list’s impact on access to medicines by evaluating a single policy aspect, such as procurement or price, which only represent a limited part of the complex purpose of the WHO model lists. ^1^

The studies that focus on broader aspects of the model list include a randomized trial showing that the distribution of essential medicines at no charge for patients compared to usual access strategies resulted in considerable increase in adherence and improvements of some surrogate outcomes.^21^ Furthermore, national legislative support is an important factor that likely maximizes the impact of the model list. When countries have not enshrined access to essential medicines in policy, decreased access and life-threatening conditions may occur.^22^ In the United States of America, several medicines listed in the WHO model list, including older, off-patent medicines, experienced uncontrolled price hikes that have disproportionately affected vulnerable populations, unlike in other countries with medicine prioritization processes where the prices of these medicines remained stable.^23^

## Challenges 

Despite these perceived successes, public health experts have criticized the model list, particularly in relation to its global role. The concerns can be broadly summarized as being about: (i) transparency and rationale of decisions by the model list expert committee;^24^ (ii) management of conflicts of interest, given applications may originate from companies producing the medicines; (iii) influence of the WHO model list on national lists; and (iv) continued tension regarding the cost of included medicines and therefore the affordability of essential medicines for different country income settings. 

### Transparency of decisions

The model list works through a process of application, review and selection. Members of the expert committee serve in their individual capacity and can accept or refute proposals made in an application. The applications and presented evidence may be interpreted as overly optimistic or even misinterpreted. Thus, to support committee members in their review of the applications, evidence is also reviewed, checked and complemented as needed by the model list secretariat, which is responsible for coordinating the decision-making process and publishing all related documents. This process has, however, posed a considerable challenge given the volume of applications received, particularly if substantial additional information is required or applications are severely flawed. As a result, applications may reflect substandard science, leading to criticism on the transparency and rationale underlying the committee’s decisions.^24^ Similar concerns on quality were directed at WHO for its guideline development process.^25^^,^^26^ Since then, guideline science has made significant advances that resulted from demands for transparency addressing shortcomings in methods and reporting.^27^^–^^29^ Through the creation of the guideline review committee and a WHO Guideline Handbook, and applying rigorous methods, significant progress has been made.^30^ Similarly, the model list secretariat is implementing a process to improve the robustness of evaluation of evidence and transparency by publishing all application documents on its website, including expert reviews and comments. The detailed rationale of the expert committee recommendations is published in the WHO Technical Report Series.^31^

### Managing conflicts of interest

Governments are paying increased attention to conflicts of interest, and applications to the model list may come from those with vested financial interests.^32^ Indeed, companies’ global market strategy may drive their desire to apply to the model list. The challenge lies in ensuring that interest is adequately managed so that the expert committee can decide on unbiased available evidence. Equally, attention to conflicts of interest among the committee’s members is critical to ensuring robust, defensible and trustworthy WHO recommendations. When selecting essential medicines, the committee’s experts can learn from WHO’s guideline recommendation development process, where in response to criticism, a process to overhaul the management of interests was implemented, mitigating risks of undue influence.^33^^,^^34^ However, even the introduction of detailed disclosure forms for WHO experts and strong mitigation strategies may not completely avoid risks of actual or perceived interests and biases. For example, in 2019, concerns about perceived experts’ conflicts of interest were among the reasons to discontinue two WHO guidelines on opioid use.^35^ While identifying and managing obvious, direct conflicts of interest in the model list process is possible, for example the exclusion of an expert who has received payments from a pharmaceutical industry, mechanisms to identify and manage indirect and less obvious conflicts of interest are more challenging, such as the influence of pervasive pharmaceutical marketing on forming an expert opinion.

### Influence on national lists

Evaluation of national lists has shown important differences in included medicines from the WHO model list, which cannot be explained by differences in the local epidemiology of diseases.^12^^,^^13^ The WHO model list is intended to support and inform national ones in diverse income settings. WHO has also developed guidance to support decision-making at a national level, and built capacity for model list development in many countries when requested.^36^

Despite WHO’s efforts, countries still have disparate processes and political considerations for listing medicines on the national list, which will ultimately affect access to essential medicines.^16^ Different political or health priorities at a national level may partly explain divergence in included medicines, but the degree of divergence suggests that processes and interpretations of the essential medicine concept and its national application are very different. In some cases, national lists have fallen far behind WHO decisions, and in others national lists are far less selective, leading to potential waste of resources. These scenarios leave many opportunities to strengthen the connection between the WHO model list and national ones.^12^ Work to catalogue and compare national lists globally and in detail for different disease areas has led to the creation of WHO’s essential medicines, a regularly updated database,^37^ which will provide opportunities to study, track and support advocacy for more model list-aligned decisions globally.

### Cost and affordability

There has been mounting controversy relating to the cost and affordability of some medicines evaluated for model list inclusion, and to the seeming contradictions by disease areas. For example, expensive hepatitis C direct-acting antivirals were included on the WHO model list, while some effective but high-priced cancer medicines have not been, despite repeated applications.^38^^,^^39^ Including cancer medicines and prioritizing those that are essential has been challenging for the model list and health systems.^40^ The 2001 inclusion criteria state that a medicine’s absolute cost is not a reason to exclude it from the model list if it meets the stated selection criteria.^1^ In the 2023 update, however, the expert committee decided not to recommend inclusion of immune checkpoint inhibitors due to their high prices and the potential diversion of relevant resources from better alternatives.^3^

Yet, the inclusion of costs as a criterion for listing challenges the focus of the model list: should the list be feasible for lower-income country setting implementation, relevant to affordable prioritization of reimbursed medicines in high-income countries, or both?^41^ While national lists will contextualize to their setting, the normative pressure for low- and middle-income countries when a medicine is listed on the WHO model list is considerable.

A recent essay has proposed a clear position on this issue: while the concept of essential medicines applies to all countries, the list as a guidance tool should refocus on low- and middle-income countries, as done initially.^42^ Providing untreated patients in low-resource settings with essential medicines is a higher priority than providing more effective or second-line essential medicines to patients already receiving some level of effective health care. Others argue that prioritizing medicines for all is a fundamental goal of government programmes regardless of economic status, and in that context the involvement of high- and upper-middle income countries in model list decisions may help lower medicine prices and facilitate technology transfer to other economies.^43^

The impact of medicines on public budgets is an important driver of health-care cost increases and a priority for all countries. In 2001, biologic medicines were less prevalent, and expensive gene therapies were not yet developed. As these new medicines may cost millions of United States dollars for single courses of treatment, the evolution in medicine price necessitates new thinking on price and affordability in the context of the sustainable development goal (SDG) target 3.8 to achieve UHC. This goal includes financial risk protection; access to quality essential health-care services; and access to safe, effective, quality and affordable essential medicines and vaccines for all by 2030.^44^ A key question is how to consider price and associated costs in model list selection, because completely disassociating price from the benefit of medicines, given spending constraints and opportunity cost, is not feasible.

## Ten visions for improvement 

In response to the challenges and research, we developed 10 visions for the future direction of the model list (Table 1) through methods detailed in Box 1. 

**Table 1:** Challenges and visions of the *WHO Model list of essential medicines*

Challenges	Vision	Description
Trust, transparency and managing conflicts of interest	Facilitating submission of high-quality applications adopting standardized contents and formats	Templates to facilitate the drafting of high-quality applications are available but are still basic. Supporting better adherence to required information and minimum standards will improve the transparency and completeness of evidence that the expert committee uses for decisions. Adherence to a quality checklist could enhance the initial screening and support WHO staff members
	Strengthened peer-review of applications	Consistent and rigorous peer-review could strengthen the process that leads to recommendations
	Criteria-based decision-making for expert committee	While the expert committee reports support rationale for decisions, documentation using a multicriteria decision-making framework would support more transparent decisions and feedback to applicants and holders with interest
	Declaration and consideration of conflicts of interest	Rigorous documentation of interests and independent review of potential conflicts and how to manage them must be always applied to support trust. Decisions in which interests may indirectly influence decisions must also be identified and managed
Connection to national lists for dissemination	Engagement of funders and/or insurance schemes	To ensure alignment between decisions and funder reality, funders' engagement in the WHO model list and national model lists processes is important
	Coordination with WHO and national health guidelines	Integration or improved connection with guidelines is needed at WHO and national levels to ensure practice guidance is available for essential medicines
	Engagement of policy-makers and impact assessment	Policy-makers should be engaged throughout the model list process, from prioritizing new medicines for consideration to implementation of decisions, including to support regulation of price of medicines and other policy decisions that influence access, which should also be measured
Cost and affordability of included medicines	Careful consideration of potential price and pathways to affordability of medicines	Price may change considerably over time. Consideration of the potential future price of a medicine may favour listing and implementation of mechanisms to improve affordability of essential medicines
	Direction of Member States in debate on low-income versus high-income level countries	WHO Member States must clarify the income-level focus of the model list, and whether the list can continue to be applicable in all income contexts, as the model list continues to consider high-priced medicines
	Model list accountability	WHO Member States should evaluate forms of responsibility or accountability to the standards proposed in the model list

WHO: World Health Organization.

Box 1 **Methods for the development of the 10 visions for the *WHO Model list of essential medicines***

The vision of the future of model lists evolved through two assessments of the country-level impact of the WHO model list, a formal qualitative study, one face-to-face consensus meeting, several online discussions and pilot testing.

Specifically, we reviewed studies about the guiding role of the WHO model list for national model lists and whether the WHO model list is aligned with the priority medicines of frontline clinicians worldwide.^12^^,^^13^^,^^19^^,^^45^^,^^46^ In our qualitative study, we explored dimensions such as conflict of interest and structured selection criteria that influence model list decisions. We piloted the criteria and how to report about them.^9^^,^^10^ Some of the vision statements had no identified empirical evidence (for example, engagement of policy-makers) and are included based on a pragmatic rationale.

The elements composing the vision were presented and discussed in a meeting convened in 2023 at WHO, where 42 key stakeholders, including 21 WHO staff members, (representing headquarters, regions and countries of WHO); six methodologists; three medicine regulators; three health economists; two representatives from the ministries and national model lists; three members of access to medicine programmes; two regulatory agency representatives; and two industry representatives, whose roles are not mutually exclusive, participated in elaborating ideas and concepts related to the future of the model list.^47^ Finally, the visions were informed by discussion in GRADE project group meetings for essential medicines but this is not an official GRADE Working Group article.^11^

Thus, although the presented visions were developed through a structured process, they are not the result of a formal consensual process but reflect the interpretation of the mentioned analyses and discussion of the authors of this paper, which was refined through multiple iterations by email. None of the authors received compensation related to the development of the vision. Some elements of these 10 visions might require additional debate and WHO direction as the model list continues to evolve.

GRADE: Grading of recommendations assessment, development and evaluation; WHO: World Health Organization.

First, to improve application quality, we propose better adherence to minimum standards of information. Applications should not have missing information and should allow using a quality checklist. Because of the large number of applications, the expert committee struggles to comprehensively review all applications. To continue reviewing medicines based on their merit rather than the quality of the applications alone, the expert committee needs further support from WHO staff members. If WHO staff members pre-screen applications using such a quality checklist, they could indicate which applications are incomplete or have insufficient quality to be taken forward to the expert committee. To fulfil such standards, at a minimum, information in any application must be based on the best available evidence from credible systematic reviews, guidelines or health technology assessments, and compare the benefits and harms of the proposed medicine(s) against any existing medicine(s) on the model list for the same indication. Placebo-controlled studies (rather than active control) should not be routinely used in the context of model list decisions. Most essential medicines were recommended because they were associated with relevant improvements in survival or decrease of serious morbidity. When essential medicines are available, offering placebo in trials is likely not justified.

Second, adherence to a quality review checklist could also strengthen peer review of applications to ensure the best available evidence is provided to the expert committee for decision-making. A large group of holders with interest in model list applications could benefit from use of standards and high-quality examples, particularly during the phase in which comments on applications are collected.

Third, rigorous evidence synthesis for model list applications does not and should not have to be performed from scratch.^7^ Efforts should be coordinated with other global health decision-making fields to minimize duplication of work.^7^ An example highlighting the importance of coordination in generating evidence that addresses global questions comes from the 2021 WHO model list application for anti-PD1 (programmed cell death protein 1) inhibitors to treat non-small cell lung cancer. In the original application, each trial was analysed in isolation from other similar trials and evidence was not meta-analysed. In the same month that the application was reviewed by WHO, a Cochrane systematic review on an identically formulated research question was published.^48^ However, the cumulated evidence from Cochrane was missed out. Other types of publications are also important, and therefore the expert committee review should take advantage of already generated multicriteria decision-making models such as the Grading of Recommendations, Assessment, Development and Evaluations (GRADE) framework – to support transparency in decisions and the rationale for its positive and negative recommendations for applications. These frameworks are usually part of previous guidelines or health technology assessments, which should be extensively searched as part of the list application process. 

Fourth, the scrutiny of potential conflicts of interests of expert committee members must continue. Independent assessment of the best available evidence is important and should be done in a manner that improves trust through rigorous management of interests. Evaluation of conflicts of applicants is less straightforward as applications can be submitted by industry. WHO could consider approaches that assess the influence of pharmaceutical lobbyists and representatives on the choice of prescriptions. Ideally, model list decisions should be informed by high-quality publicly funded trials, particularly large head-to-head trials that compare multiple treatments for a condition. 

Fifth, to improve access more effectively, essential medicines must be affordable components of a UHC system. For model lists to cover gaps in access to medicines for priority conditions, health system funders and insurers should be engaged in decision-making processes to ensure a grounding in affordability. While linkages to strategies already exist for global access such as pooled procurement, tiered pricing and voluntary licencing, access to medicines in countries is ultimately contingent on national pharmaceutical and health insurance policies to enable affordable access. A periodic feedback system to WHO that explicitly describes if the model list is moving too far ahead of countries’ capacities to implement the recommendations should be considered.

Sixth, we also envision rapid coordination between guideline developers and model lists in a reciprocal relationship, both at WHO and in countries. Some WHO departments effectively link their guidelines with the model list, but this linkage needs to be generalized to address gaps across different disease areas. For example, a simultaneously organized guideline and model list meeting in 2002 led to an important collaborative WHO guideline and model list for antiretrovirals, driving forward the HIV access agenda.^49^ However, the timing of WHO guidelines and the model list update sometimes misalign, causing a two-year gap or more in recommendations. A possible remedy is to accept model list applications derived from WHO guidelines annually instead of biennially.

Seventh, engagement with evidence-informed policy-making experts to ensure that inclusion decisions are translated into national lists and national priorities and policy decisions. Implementing access to medicines requires experts in implementation, a scarce human resource in most settings. Health decisions, including decisions on access to and coverage of essential medicines, are inherently political and built on policy decisions. Working with the evidence-informed policy-making field will help align recommendations for medicines with knowledge translation (for example, dissemination and consultation) for policy-makers. This approach will ultimately influence field implementation and access to medicines, because national policy-makers are the ones who shift resources and set policies on medicines, and create access through reimbursement thresholds and laws for coverage of medicines. They also may implement levers to regulate or influence price by industry. Thus, to provide countries with effective model lists, building greater political endorsement of the role of the model list is necessary.

Eighth, the view that the model list cannot be applicable to all settings and should focus on the needs of low- and middle-income countries has merits.^42^ However, possibly only a few model list recommendations do not apply in low-resource contexts. The root cause for the large differences across medicine prioritization in low- and middle-income countries should be investigated by researchers and recognized by policy-makers as a gap in achieving UHC; strategies to speed dissemination of and equitable access to the optimal therapies should be carefully sought. As the international health community, including academia and WHO, continue assessing the cost and affordability of essential medicines, we suggest that consideration of the potential future price of a medicine may facilitate listing and subsequent implementation of mechanisms to improve affordability of essential medicines.

Ninth, as the expert committee continues to consider high-priced medicines, WHO Member States must clarify the income-level the model list should focus on, and whether that level can continue to be applicable in all income contexts.

Tenth, while the model list does not have a contractual character for countries, it still has merits as a potential constitutional benchmark to be kept at the centre of countries’ legislations. A constitution that recognizes that not all medicines are equal and that those that are more important should be prioritized, creates a legitimate expectation that UHC can be attained.

## Prospects

After its 2023 meeting, the expert committee noted that the procedure for updating the model lists had only been revisited once since the first model list in 1977, and recommended WHO to consider initiating a process to reassess the procedure for updating the model list.^3^ Here we have outlined a strategic vision to update both the criteria used for model list decision-making and the process.

Despite the challenges enumerated, access to essential medicines through policy that is based on the model list and national uptake remains a social global priority. Essential medicines are a focus of UHC and serve as an important focal point for ensuring medicine coverage and access in many settings.^13^ Ensuring the model list continues to meet modern day challenges is a critical priority to maintain its relevance and trust among WHO’s normative and standard setting tools. Pharmaceutical spending accounts for an average of 15% of health system expenditures in many countries and is continuing to grow; ensuring resources are spent on the highest value essential medicines is therefore imperative.^50^

For the model list to continue improving access to essential medicines through country-level policies, the procedure for the model list must evolve to meet current challenges and address future ones.^3^ Our proposal is to accomplish this goal through reassessing the 2001 criteria and procedures for updating the model list; strengthening links with other health decision-makers within and outside of WHO to harmonize health guidance and listings; improving the quality of model list applications; and enhancing capacity-building for developing and updating of national lists. 
